# Co‐Producing Evidence‐Based Care: Nurses' and Patients' Lived Experiences in Long‐Term Condition Management

**DOI:** 10.1111/jan.70127

**Published:** 2025-08-05

**Authors:** Jude Ominyi, Andrew Clifton, Uchenna Chima

**Affiliations:** ^1^ School of Health Sciences University of Suffolk UK; ^2^ School of Nursing and Allied Health Newman University Birmingham UK

**Keywords:** co‐production, evidence‐based practice, interpretative phenomenological approach, knowledge implementation, long‐term conditions, nursing practice, person‐centred care

## Abstract

**Aim:**

To explore the lived experiences of nurses and patients co‐producing evidence‐based care for long‐term conditions, and to understand how they make sense of this process within relational, emotional and organisational contexts.

**Design:**

A qualitative study using the Interpretative Phenomenological Approach.

**Methods:**

Semistructured interviews were conducted with 20 participants, comprising 11 registered nurses and 9 adult patients living with at least one Long‐Term Condition. Participants were recruited from primary and secondary care settings across the Midlands, England. Data were collected between February and August 2023 and analysed using Interpretative Phenomenological Approach's iterative and inductive framework.

**Results:**

Five experiential themes were identified: (1) weaving together different knowledges, (2) the relational foundations of co‐production, (3) organisational pressures and misalignments, (4) shifting identities and power dynamics and (5) emotional and ethical complexity in co‐producing care. Participants described co‐production as a deeply relational and negotiated process, shaped by trust, vulnerability and shared decision‐making.

**Conclusion:**

Co‐producing evidence‐based care in Long‐Term Condition management involves more than implementing guidelines. It is a relational, emotional and contextual practice that requires shared interpretation of evidence, deep listening and responsiveness to individual lives. Findings suggest a need to reframe evidence‐based practice as a co‐creative process grounded in relational ethics and contextual awareness.

**Impact and Implications:**

Findings emphasise the centrality of relational competence and organisational flexibility in enabling co‐produced care. Findings call for educational and policy reforms that value emotional labour, professional humility and patient knowledge as essential to evidence‐based nursing. Internationally, this work provides a grounded model for integrating person‐centred approaches into chronic care delivery and policy.

**Contribution to the Wider Global Clinical Community:**

The study offers a relational model of evidence‐based practice that moves beyond protocol‐driven care to one shaped through dialogue, empathy and contextual negotiation, offering practical insights for transforming professional roles and health systems globally.

**Patient and Public Involvement:**

Patient representatives contributed to study design, development of interview guides and interpretation of findings to ensure alignment with lived experiences.

**Reporting Method:**

This study follows the SRQR guideline.

**Summary**

- What does this paper contribute to the wider global clinical community?
  ○ This study reconceptualises evidence‐based practice as a co‐produced, relational practice grounded in shared expertise and lived experience to support more person‐centred care.
  ○ It highlights the value of trust, empathy and emotional labour in managing long‐term conditions collaboratively, offering practical insights for improving long‐term condition management globally.
  ○ Findings offers practice and policy insights for building care systems that are responsive to patients' lives rather than metrics alone.

## 1 Introduction

Evidence‐based practice (EBP) is widely accepted as central to delivering high‐quality, safe and person‐centred nursing care (Melnyk et al. 2014; Saunders et al. 2019). EBP supports informed healthcare decision‐making through the integration of the best available research evidence, clinical expertise and patient preferences (Ominyi, Nwedu, et al. 2025). More recently, EBP has been reinterpreted through the perspective of implementation science, where it is understood less as the direct application of research findings and more as a negotiated and relational process embedded within the complexities of clinical, interpersonal and organisational life (Greenhalgh et al. 2004; Ominyi and Alabi 2025a).

Policy and educational initiatives continue to promote EBP across healthcare settings, yet many nurses face persistent barriers to integrating it into routine clinical practice (Rycroft‐Malone et al. 2002). Frontline practitioners frequently describe constraints such as limited time, hierarchical structures, insufficient preparation and organisational resistance (Geerligs et al. 2018; Kajermo et al. 2010). These challenges reflect more than practical obstacles. They raise important questions about the value placed on different kinds of knowledge, the distribution of expertise and the organisational conditions that shape whose knowledge counts in practice (Ominyi and Alabi 2025a). Alongside these developments, co‐production has gained increasing attention within healthcare policy. Rather than viewing patients as passive recipients, co‐production advocates for their active engagement in the design, delivery and evaluation of care (Palmer et al. 2019; Realpe and Wallace 2010). The principles underpinning co‐production resonate with person‐centred and relationship‐centred approaches to nursing, which have long emphasised mutual respect, shared decision‐making and relational engagement (McCormack and McCance 2006). In the context of long‐term condition (LTC) management, where trust, sustained interaction and self‐management are vital, co‐production offers a particularly relevant model (Coulter et al. 2015), by offering not only a philosophy of care but a practice of evidencing that foregrounds collaboration, mutual learning and epistemic justice (England 2022). Despite these conceptual alignments, limited empirical research has explored how EBP and co‐production interact in day‐to‐day nursing practice. While policy discourse increasingly champions both, few studies examine how nurses and patients work together to negotiate and apply diverse forms of knowledge; scientific, experiential and contextual, within the real‐world constraints of relational and organisational settings. This study addresses this important gap by investigating the lived experiences of nurses and patients who engage in co‐producing care in the management of LTCs.

## 2 Background

Embedding evidence into clinical practice has remained a central goal of healthcare reform, particularly in the management of chronic conditions such as type 2 diabetes, cardiovascular disease, and chronic obstructive pulmonary disease (COPD), which together account for a substantial proportion of healthcare use in the United Kingdom (Department of Health and Social Care 2023; World Health Organization 2023). EBP is widely regarded as a key strategy for improving patient safety, ensuring consistency in care and supporting collaborative decision‐making through the integration of research evidence, clinical judgement and patient preference (Melnyk et al. 2014). In the context of long‐term condition care, however, EBP is increasingly understood not as the straightforward application of clinical guidelines, but as a negotiated, socially embedded practice shaped by patient values, professional judgement and organisational conditions (Gagliardi et al. 2016; Robert et al. 2022). Nurses frequently encounter barriers to this work, including organisational inertia, time pressure and limited autonomy, which together create tensions between the ideals of evidence‐based care and the constraints of everyday clinical practice (Chan et al. 2021; Hakkennes and Green 2006).

Co‐production has increasingly gained attention as a model that seeks to rebalance traditional power dynamics in healthcare by recognising the lived knowledge, values and experiences of patients. Rather than positioning patients as passive recipients of care, it promotes genuine partnership and shared ownership in care planning and delivery (Palmer et al. 2019; Robert et al. 2022). This aligns closely with person‐centred nursing, where relational and ethical engagement forms the basis of practice (McCormack and McCance 2006). For individuals living with long‐term conditions who often develop deep, embodied knowledge of their illness, co‐production offers a meaningful way to integrate this expertise into clinical decision‐making (Entwistle and Cribb 2013).

However, despite the theoretical compatibility between EBP and co‐production, they are often treated as distinct practices in both research and clinical settings. Traditional EBP frameworks tend to privilege evidence derived from randomised controlled trials and systematic reviews, often marginalising contextual, cultural and experiential knowledge (Bull et al. 2022; Ominyi and Alabi 2025a; Ominyi and Alabi 2025b). This presents a challenge for nurses tasked with individualising care while managing the relational complexities of LTC management (Clarke et al. 2020; Hughes and McCormack 2024).

Emerging scholarship has called for a broader understanding of evidence in clinical decision‐making. Patient narratives, socio‐cultural contexts, financial constraints and intuitive, embodied knowledge are increasingly recognised as essential elements of truly person‐centred care (Gagliardi et al. 2016; Robert et al. 2022). Yet, relatively little empirical work has explored how nurses and patients jointly make sense of and work with these diverse forms of knowledge, particularly within the emotionally and relationally demanding context of long‐term condition care.

Existing studies frequently focus on either EBP or co‐production, without examining the relational, organisational and ethical conditions necessary to support their integration (Bull et al. 2022; Chan et al. 2021). While significant attention has been given to the barriers involved in applying clinical guidelines or involving patients in decision‐making (Bull et al. 2022; Robert et al. 2022; Elwyn et al. 2012), there remains limited exploration of how evidence is constructed and shared within nurse–patient relationships. Even fewer investigations consider the influence of leadership, time allocation and documentation systems on these processes. These gaps highlight the need for research that explores how EBP and co‐production converge in the realities of everyday nursing practice. This study addresses this need by focusing on how nurses and patients engaged in LTCs management experience and make sense of co‐producing care.

## 3 The Study

### 3.1 Aim

This study aimed to explore the lived experiences of nurses and patients and to understand how they make sense of co‐producing care through evidence‐based practice in the management of long‐term conditions. The study was guided by the following research questions:

1. How do nurses and patients experience and make sense of co‐producing care in the management of long‐term conditions?
2. What meanings do nurses and patients attribute to their experiences of working with evidence within co‐produced care encounters?
3. How do nurses and patients experience and make sense of the relational, emotional and organisational dimensions that shape co‐produced, evidence‐based care?

## 4 Method

### 4.1 Study Design

We adopted the Interpretative phenomenological approach (IPA) (Smith et al. 2009; Smith and Nizza 2022). We considered IPA appropriate for its capacity to support a rich, idiographic examination of how participants make sense of significant health‐related experiences (Smith and Nizza 2022). IPA privileges the exploration of how individuals interpret their lived experiences within their relational and contextual worlds (Eatough and Smith 2017; Pietkiewicz and Smith 2014). The choice of IPA was particularly appropriate given the complexity and relational nature of co‐produced care in LTC management. As participants reflected on how they navigated clinical guidelines, personal experience and interpersonal dynamics, IPA provided the conceptual space to examine these nuanced, emotionally resonant narratives without reducing them to predefined categories. The interpretative, double hermeneutic process at the heart of IPA allowed the researchers to engage deeply with how participants made sense of care encounters and the role of evidence within them (Smith and Osborn 2007). The use of IPA in health research continues to grow, particularly in areas where deep insight into patient and practitioner experience is required (Sloan and Bowe 2014).

### 4.2 Study Settings

Participants were recruited from a range of healthcare services located in the East Midlands region of England. These services included general practice surgeries and hospital outpatient clinics that specialise in the management of LTCs, such as type 2 diabetes, COPD, hypertension, heart failure and chronic kidney disease. Settings were purposefully selected to reflect variation in service organisation, patient populations and clinical focus, recognising that the delivery of care and the experience of EBP are influenced by the organisational and relational contexts in which they occur. Including both community‐based and hospital‐based services ensured that the study captured diverse perspectives on how EBP and co‐produced care are practised and experienced in different clinical environments.

Access to participants was facilitated through clinical care teams working in these settings. Clinical Leads were informed about the study and shared recruitment materials with eligible individuals, helping to ensure that the introduction to the study was integrated within existing care relationships. This approach respected the role of clinical teams in supporting patient engagement and upheld ethical standards of voluntary participation based on fully informed consent. The selection of settings and the approach to recruitment were intended to maximise the ecological validity of the study, ensuring that the findings would reflect the complexity and variability of real‐world clinical practice (Eatough and Smith 2017; Pietkiewicz and Smith 2014).

### 4.3 Sampling and Recruitment

Purposive, criterion‐based sampling was used, consistent with the methodological principles of IPA, which seeks depth and richness of individual accounts rather than statistical representation (Pietkiewicz and Smith 2014; Smith and Osborn 2007). Two groups of participants were invited: (1) adult patients living with at least one long‐term condition who had experience of engaging in collaborative care planning, and (2) registered nurses actively involved in the management of LTCs within community or hospital services.

Eligibility criteria for nurses included current registration with the Nursing and Midwifery Council, employment in a clinical role focused on chronic disease management and regular involvement in shared decision‐making with patients. Patients were required to be aged 18 years or older, have a diagnosis of at least one long‐term condition and possess the ability to participate in an interview in English.

Recruitment materials, including an invitation letter and participant information sheet, were distributed by the clinical care teams. The materials clearly explained the study purpose and the meaning of key terms such as ‘co‐produced car’ and ‘evidence‐based practice’, ensuring that potential participants could make an informed decision about involvement. The information sheet was reviewed with the support of Patient and Public Involvement (PPI) contributors to promote clarity, accessibility and sensitivity to the experiences of patients managing long‐term conditions.

Recruitment continued until a sample that reflected a range of experiences and backgrounds had been achieved. Eleven nurses and nine patients consented to participate. Although IPA studies often work with smaller samples, this number was consistent with the idiographic focus of IPA and the aim to support both individual depth and the possibility of cross‐case analysis where appropriate (Eatough and Smith 2017; Smith and Osborn 2007). The emphasis was not on reaching data saturation, which is not a goal in IPA, but on ensuring that the accounts collected offered sufficient richness and variation to support a detailed interpretative analysis [30–31]. The final sample was judged to have achieved analytic sufficiency, providing diverse and meaningful insights into how nurses and patients make sense of co‐producing care through EBP in the management of LTCs.

### 4.4 Data Collection

Data were collected through semistructured, one‐to‐one interviews using two carefully developed interview guides: One for patients and one for nurses (see Tables 1 and 2). The guides were co‐designed with input from PPI contributors, ensuring that the questions were accessible, respectful and reflective of participants' lived experiences (Sloan and Bowe 2014). The semistructured approach allowed participants the flexibility to direct the conversation towards issues they found meaningful while enabling the researchers to explore specific areas of interest related to co‐producing care and working with evidence. Gentle prompts were employed when needed to deepen the exploration and invite participants to elaborate, ensuring that their accounts remained central to the dialogue.

**TABLE 1:** Nurse interview guide.

Interview question	Prompt questions
1. Can you describe your specific role in supporting patients with long‐term conditions?	1.1 What conditions do you most frequently manage? 1.2 What is the typical structure of your consultations? 1.3 How do you build rapport with your patients?
2. What does co‐production of care mean to you in your practice?	2.1 How do you involve patients in their care planning? 2.2 Can you share an example of a co‐produced decision? 2.3 Are there situations where co‐production feels more difficult? Why?
3. How do you use evidence when working with patients to plan care?	3.1 Do you rely on specific guidelines (such as NICE)? 3.2 How do you balance clinical guidelines with patient preferences? 3.3 Have patients ever challenged or questioned the evidence you presented?
4. How do patients contribute their own knowledge or experiences to the decision‐making process?	4.1 Do you see patients as experts in their own conditions? 4.2 Can you recall a time when patient input changed your clinical recommendation? 4.3 How do you validate patient narratives or embodied knowledge?
5. What organisational, emotional or relational factors influence your ability to engage in co‐produced, evidence‐based care?	5.1 How does time impact your ability to involve patients? 5.2 Are there emotional or interpersonal dynamics that help or hinder co‐production? 5.3 Does your team or service context support this way of working? 5.4 What policies, tools or structural factors shape your practice?

**TABLE 2:** Patient interview guide.

Key questions	Prompts
1. Can you tell me about your experience of living with your long‐term condition?	1.1 How was your condition first diagnosed? 1.2 How has it affected your daily life? 1.3 What symptoms have been most challenging to manage? 1.4 How has your experience changed over time?
2. How have healthcare professionals, especially nurses, been involved in managing your condition?	2.1 What role does the nurse play in your care? 2.2 How often do you see the nurse? 2.3 Do you feel your concerns are understood and addressed?
3. What does it mean to you to be involved in decisions about your care?	3.1 Can you describe a time when you felt involved in making a care decision? 3.2 Were your knowledge and preferences taken into account? 3.3 What helped or hindered your involvement?
4. What kinds of information or evidence have you used when making care decisions?	4.1 Did the nurse discuss research or treatment guidelines with you? 4.2 How have you brought your own experiences or knowledge into care discussions? 4.3 Have you used other sources of information, such as online resources or support groups?
5. What supports or challenges have you encountered when co‐producing your care?	5.1 What has helped you feel more confident or included? 5.2 Have you faced any barriers, such as communication difficulties or time constraints? 5.3 How could care be improved to better include your voice?

While the interview guides did not directly separate relational, emotional and organisational dimensions, these aspects surfaced naturally in participants' narratives. For example, discussions of co‐production frequently led to reflections on trust, empathy and emotional safety. Similarly, questions about barriers to engagement often elicited accounts of time pressures, structural constraints and organisational routines. In the nurse guide, Question 5 was designed to invite direct consideration of the organisational context and was subsequently expanded to more explicitly explore emotional and relational dynamics that influence co‐produced care. This way, the data that addressed research question 3 were largely generated inductively and were interpreted through participants' embedded accounts of their day‐to‐day clinical experience (Eatough and Smith 2017; Smith and Nizza 2022).

Interviews were conducted between February and August 2023. Participants were offered a choice of interview modes based on their preferences and practical considerations. Thirteen interviews were carried out face‐to‐face, either at clinics, community health centres or participants' homes, while three were conducted via Zoom, and four by telephone. Where possible, participants were encouraged to use video to foster a more conversational and connected atmosphere, although some opted for audio‐only to accommodate privacy and comfort. Interviewing modes were adapted flexibly to support a safe and comfortable environment for all participants (Palmer et al. 2019).

The interviews were conducted by the first and second authors, both experienced qualitative researchers with backgrounds in nursing. Each interview lasted between 60 and 120 min. All interviews were audio‐recorded with participant consent, transcribed verbatim and anonymised during transcription to protect confidentiality. Care was taken to preserve the richness of the participants' language and to attend closely to tone, pauses and emphasis, recognising these as integral to the interpretative process in IPA (Eatough and Smith 2017).

Demographic details, including age, gender, clinical role (for nurses), condition type (for patients) and relevant cultural or personal identity markers, were collected at the start of each interview. This contextual information supported the idiographic focus of the analysis and enabled a more nuanced interpretation of participants' experiences (Eatough and Smith 2017).

### 4.5 Data Analysis

Data were analysed using IPA, following the six‐stage, idiographic and inductive process described by Smith et al. (Eatough and Smith 2017) and developed further by Smith and Nizza (Smith and Nizza 2022). This approach was selected to support a close, psychologically informed exploration of how nurses and patients make sense of co‐producing evidence‐based care in the context of LTCs.

#### 4.5.1 Reading and Rereading

Each transcript was read multiple times to support immersion and develop a sustained, empathic engagement with the participant's experiential world. Attention was given to subtle shifts in tone, pace, metaphor and emphasis that signalled underlying emotional or psychological content.

#### 4.5.2 Initial Noting and Experiential Statements

Detailed initial noting was conducted in three forms: Descriptive (focused on content), linguistic (attending to rhythm, metaphor and hesitancy) and conceptual (highlighting patterns of meaning or interpretative possibilities). These notes informed the development of experiential statements, short analytic units that expressed what the participant appeared to be working through, discovering or reflecting upon. These statements were grounded in participants' language but reached towards deeper psychological meaning. For example, a patient who said, ‘I've lived with diabetes for 15 years. I know my body’, was interpreted as asserting experiential authority and challenging traditional hierarchies of clinical evidence.

#### 4.5.3 Clustering Experiential Statements

Experiential statements were grouped into meaningful clusters that captured coherent lines of meaning. These formed the basis for personal experiential themes. In one instance, a nurse's transcript yielded 89 experiential statements, which were clustered into themes such as relational continuity, navigating evidence hierarchies, cultural sensitivity and organisational tension.

#### 4.5.4 Developing Personal Experiential Themes

Each participant's narrative was synthesised into three to five overarching personal experiential themes, preserving the idiographic focus of IPA. These themes reflected what was most psychologically and relationally significant to the individual, based on their account of co‐producing evidence‐based care. Themes such as ‘knowing my own body’, ‘being heard’ or ‘navigating fragmented care’ captured the layered complexity of these accounts.

#### 4.5.5 Cross‐Case Analysis and Development of Group Experiential Themes

Themes were compared across transcripts to identify patterns of similarity and difference. Group experiential themes were defined as those occurring in at least half of the transcripts. These included recurring challenges related to the negotiation of different forms of knowledge, the emotional labour of collaborative care and the influence of structural constraints such as time, organisational culture and documentation systems. Data that did not recur across participants were not excluded. Instead, they were retained as analytically valuable deviations and used to enrich the interpretation of dominant patterns. This allowed for a more nuanced and layered account of experience. For instance, although only a few nurses discussed tensions with digital documentation systems, these insights deepened the contextual understanding of co‐production.

#### 4.5.6 Interpretative Write‐Up and Conceptual Engagement

The final write‐up involved developing interpretative narratives around the group and personal themes, supported by illustrative participant quotations. During this phase, two conceptual frameworks were introduced to aid interpretation: McCormack and McCance's (McCormack and McCance 2006) Person‐Centred Nursing Framework and Rycroft‐Malone's (Rycroft‐Malone 2004) Contextual Model of EBP. These frameworks were not used to guide early analysis or shape coding, and their inclusion does not conflict with the philosophical foundations of IPA. Their integration was limited to the postanalytic stage, when the research team observed that participants' experiential accounts bore strong resonance with the relational and contextual dimensions outlined in these models.

McCormack and McCance's framework helped to elaborate how emotional engagement, professional competence and therapeutic presence shaped co‐production from the perspectives of nurses and patients. Rycroft‐Malone's model provided a useful interpretive lens for exploring how organisational culture, facilitation and different forms of evidence interacted within care environments. These frameworks were not used to classify or quantify the data. Instead, they supported deeper conceptual reflection and helped articulate theoretical insights embedded in participants' accounts. This approach is consistent with IPA's allowance for later‐stage engagement with relevant theory, particularly where doing so enriches the contextual and interpretative depth of findings (Eatough and Smith 2017; Smith and Nizza 2022). Tables 3 and 4 are presented below to summarise the analytic process and illustrate how conceptual frameworks were used to support interpretation.

**TABLE 3:** Key steps in IPA analysis.

Stage	Description
Reading and rereading	Transcripts were read multiple times to support immersion. Initial notes captured emotional tone, linguistic features and early conceptual insights.
Initial noting and experiential statements	Descriptive, linguistic and conceptual notes informed experiential statements reflecting psychological and relational meaning.
Clustering experiential statements	Experiential statements were grouped into thematic clusters, forming initial personal experiential themes.
Personal experiential themes	Each narrative was synthesised into 3–5 personal themes, preserving idiographic integrity.
Cross‐case analysis	Group themes were developed from recurring personal themes. Divergent cases were retained to enrich interpretation.
Interpretative write‐up	Illustrative quotations were used to support final interpretation. Conceptual frameworks were drawn on postanalysis to reflect on relational and contextual patterns.

**TABLE 4:** Framework‐linked concepts used to support interpretation.

Framework	Conceptual element	Example of relevance to data
McCormack and McCance (2006)	Mutual respect and knowing the patient	Nurses described how sustained relationships and emotional presence supported co‐production.
McCormack and McCance (2006)	Professional competence and relational engagement	Themes of compassion, responsiveness and ethical care emerged strongly across accounts.
Rycroft‐Malone (2004)	Context and facilitation	Nurses described time pressures, documentation burdens and local norms that shaped co‐production practices.
Rycroft‐Malone (2004)	Interaction of evidence types	Participants reflected on balancing clinical guidelines, personal experience and patient preferences.

These frameworks supported the interpretative depth of the final write‐up and helped convey the relational and contextual intricacies of co‐produced evidence‐based care.

### 4.6 Reporting Method

This study adhered to the SRQR guidelines (O'Brien et al. 2014), including explicit reporting of researcher positionality, data saturation strategy and participant–researcher relationship.

### 4.7 Rigour and Reflexivity

Rigour in this study was upheld by adopting strategies that align with the philosophical foundations of IPA. A clear audit trail was maintained throughout the research process, documenting all stages of data collection and analysis. This included reflexive journals kept by the lead researcher and regular analytic memos that recorded interpretive decisions and reflections on the evolving analytic process. These practices ensured that the development of experiential themes was traceable and grounded in the original data. Rather than seeking consensus through triangulation, different perspectives within the research team were used to critically discuss interpretations. Each researcher analysed a subset of transcripts independently, and analytic meetings were held to reflect on points of convergence and divergence. These discussions did not aim to eliminate subjectivity but to recognise it as an inherent part of the double hermeneutic process central to IPA, where researchers interpret participants' interpretations of their experiences (Sloan and Bowe 2014; Larkin, Eatough, and Osborn 2011). This approach supported reflexive interpretation while maintaining fidelity to participants' accounts.

The study did not employ member checking, in line with the view that in IPA the aim is not validation by participants but to develop a rich, interpretative understanding of how participants make sense of their experiences (Smith and Nizza 2022). Participants' meanings were treated as situated, dynamic and contextually embedded, and the interpretative process was reflexively acknowledged as an active engagement rather than a neutral reflection. Reflexivity was an integral part of the research process. The lead researcher, a nurse academic with prior clinical experience in long‐term condition management, maintained a reflexive journal to record preconceptions, assumptions and emotional responses throughout the study. Regular discussions with co‐researchers provided a space for critical reflection on how the research team's disciplinary backgrounds, values and experiences shaped the interpretation of the data. This practice helped to sustain a balance between empathic engagement with participants' accounts and critical self‐awareness. Thick description was used to illustrate the themes, with verbatim quotes selected to capture the richness and complexity of participants' lived experiences (Eatough and Smith 2017). Finally, transparency was enhanced through detailed reporting of the study context, sampling strategy and analytic procedures, providing readers with sufficient information to assess the trustworthiness of the study. These strategies, rooted in IPA's methodological traditions, supported the production of an interpretative account that is credible, coherent and grounded in participants' lived realities.

### 4.8 Patient and Public Involvement

Patient voices were included throughout the study design and delivery using a co‐productive approach. Patient representatives were involved in the development of the interview topic guides, participant information sheets and consent forms. Their input helped ensure that the study materials were accessible, relevant and sensitive to the lived realities of managing LTCs. Two patient contributors with LTCs participated in practice interviews to refine the flow, tone and clarity of the questions. Feedback from these practice interviews was collated and discussed among the wider research team to enhance the study's credibility and acceptability. Patient representatives also advised on the recruitment strategy and identified ways to make the interview process more inclusive and less burdensome for participants. To further support credibility and transparency, findings were shared with patient representatives through a structured member‐checking process. Their reflections were used to confirm the resonance and accuracy of the emergent themes and to strengthen the interpretation of key insights.

### 4.9 Ethical Considerations

The study was conducted in accordance with the guidelines of the Declaration of Helsinki and was approved by the University of Beds Research Ethical Approval (Reference ID: UoB/00184; Date of approval: 07.09.22), ensuring compliance with national regulations. One amendment was subsequently submitted and approved to accommodate expanded recruitment strategies and clarify the use of remote interviews. Verbal consent was gained prior to the start of each interview, in addition to the written informed consent obtained beforehand. All interview data were anonymised during transcription, and pseudonyms were assigned to all participants. De‐identified transcripts were reviewed by the second and third researchers to support interpretive rigour while maintaining participant confidentiality. Given the diversity of the sample, ethical consideration was given to power dynamics. Efforts were made to create a psychologically safe interview environment, with an emphasis on voluntary participation and withdrawal rights.

## 5 Findings

### 5.1 Characteristics of the Participants

The sample included nurses and patients with a wide range of clinical backgrounds, years of experience and LTCs. This diversity supported the generation of nuanced insights into how evidence‐based care is co‐produced across different relational, organisational and experiential contexts. Table 5 presents a summary of the demographic characteristics of the nurse participants, while Table 6 provides an overview of the patient participants' profile.

**TABLE 5:** Nurse participants.

Characteristics	Number (*n* = 11)
Gender	
Male	3
Female	8
Age profile	
30–39 years	2
40–49 years	4
50+ years	5
Years of experience	
< 15 years	3
≥ 15 years	8
Highest educational qualification	
Bachelor's Degree	4
Master's Degree or working towards one	7
Clinical specialty	
Diabetes care	3
Respiratory care (COPD, asthma)	2
Cardiovascular care (heart failure, hypertension)	3
Renal care (chronic kidney disease)	2
General LTC management	1
Practice setting	
Community‐based general practice	6
Hospital outpatient services	5

**TABLE 6:** Patient participants.

Participant ID	Age (Years)	Gender	Primary LTCs
P01	52	Female	Type 2 diabetes
P02	65	Male	Chronic Obstructive Pulmonary Disease
P03	68	Female	Hypertension
P04	58	Male	Chronic kidney disease
P05	71	Female	Heart failure
P06	66	Male	Type 2 diabetes, Hypertension
P07	60	Female	Chronic Obstructive Pulmonary Disease
P08	74	Male	Type 2 diabetes
P09	76	Female	Hypertension

### 5.2 Overview of Findings

The five overarching experiential themes identified through IPA reflect the lived experiences of nurses and patients as they co‐produced evidence‐based care. These themes do not stand in isolation but are interwoven across personal, organisational and relational dimensions. They highlight the negotiated and sometimes contested processes through which formal evidence, lived knowledge and care relationships are enacted in everyday practice. Table 7 outlines the themes, subthemes, and provides illustrative quotations drawn from participant interviews, offering an initial glimpse into the interpretative depth that follows.

**TABLE 7:** Themes, subthemes and illustrative quotes.

Themes	Subthemes	Illustrative quotes
Weaving together different knowledges	Evidence as guidance, not a prescription	‘The guidelines give us the foundation, but it is not enough. Patients live complex lives that don't always match what the evidence says should happen […] We have to work around that, not force it’ (Nurse 05).
	Patients' lived experience as legitimate evidence	‘I've lived with this condition for nearly twenty years. I know the small signs my body gives me […] That knowledge matters, even if it's not in a study’ (P07).
The relational foundations of co‐production	Tensions between guidelines and real life	‘They told me to exercise thirty minutes a day. It sounds simple until you factor in that I've got arthritis and I'm on a fixed income’. (P04)
	Trust as a precondition for engagement	‘Trust isn't given automatically. It's earned through every conversation, every moment you genuinely listen’ (Nurse 06).
Organisational pressures and misalignments	Relational labour and professional vulnerability	‘It's not easy to admit when you don't have all the answers. But I find that patients respond better when you show you're human too’ (Nurse 02).
	Time as the missing ingredient	‘Appointments are fifteen minutes. How do you build trust, have a real conversation and make a shared decision in that time?’ (Nurse 08).
	Metrics over meaning	‘You end up chasing numbers… blood pressure targets, cholesterol levels… but miss the person’ (Nurse 10).
	Variability across settings	‘In the community clinic, we're given a bit more breathing room. In hospital, it's target‐driven […] You do what you can, but the structure makes it harder’ (Nurse 01).
Shifting identities and power dynamics.	Patients as active partners	‘Before, it felt like they just told you what to do. Now, they ask me what's realistic. It feels more like I have a say’ (P08).
	Nurses redefining expertise	‘I used to think my job was to have the answers. Now, I see it as helping the patient find what works for them’ (Nurse 07).
Emotional and ethical complexity in co‐producing care.	Navigating emotional landscapes	‘You're not just dealing with blood sugars or blood pressure. You're dealing with fear, frustration, hope. Ignoring that would be bad care’ (Nurse 04).
	Ethical tensions in shared decision‐making	‘There are times when I know the evidence says one thing, but the patient's choice goes another way. It's hard, but respecting that choice is part of the partnership’ (Nurse 11).

The individual themes and subthemes are, in turn, presented here below.

### 5.3 Weaving Together Different Knowledges

Participants consistently described co‐production not as the simple application of evidence, but as the dynamic weaving together of multiple knowledges: clinical guidelines, professional experience and the lived expertise of patients. Rather than following protocols rigidly, nurses and patients negotiated care decisions in context, adjusting formal recommendations to meet the realities of everyday life. This required a relational approach to knowledge, where expertise was shared and the boundaries between evidence and experience were fluid. The subthemes below illustrate how participants framed evidence as flexible, recognised the legitimacy of lived experience and navigated tensions between guidelines and the complexities of daily living.

#### 5.3.1 Evidence as Guidance, Not a Prescription

Many nurses viewed evidence‐based guidelines as essential reference points, but not as rigid prescriptions. Instead, they treated evidence as something to be interpreted and adapted to suit individual circumstances.

…the guidelines give us the foundation, but it is not enough […] Patients live complex lives that don't always match what the evidence says should happen…We have to work around that, not force it (Nurse 05).

This shows that clinical practice involves adapting evidence through judgement and dialogue, rather than simply following set pathways. Patients echoed this sentiment, describing how care became meaningful only when practitioners attended to their personal circumstances:

I know what the textbooks say, but I'm not a textbook case…they [nurses] have to listen to what's happening in my real life, or the advice just won't work (P02).

This reciprocity highlights a central insight affirming that co‐produced care becomes possible when professionals move beyond procedural fidelity and focus on fit, feasibility and responsiveness.

#### 5.3.2 Patients' Lived Experience as Legitimate Evidence

Generally, patients shared a deep awareness of their own bodies and conditions. Their narratives reflected a sense of embodied expertise that, while often unacknowledged by formal systems, was integral to their self‐management.

…I've lived with this condition for nearly twenty years…I know the small signs my body gives me…things a guideline doesn't cover. That knowledge matters, even if it's not in a study (P07).

Such reflections show that patients bring a form of experiential evidence to the clinical encounter, one that is specific, situated and hard‐won. When nurses recognised this knowledge, it became a powerful resource for shared care planning. As one nurse reflected:

…I learned early on that patients often know more about living with their condition than I do. It's humbling but also eye‐opening. Their knowledge fills in the gaps that evidence leaves (Nurse 03).

These exchanges suggest that valuing lived experience is not simply about patient empowerment; it enhances the epistemic quality of care by integrating knowledge forms that are otherwise invisible to standardised evidence models.

#### 5.3.3 Tensions Between Guidelines and Real Life

Participants often spoke about the friction that arises when clinical recommendations clash with the complex textures of patients' lives. These tensions were not rare anomalies but everyday realities requiring creativity and compromise.

They told me to exercise thirty minutes a day. It sounds simple until you factor in that I've got arthritis and I'm on a fixed income. Walking around the block costs nothing, but a gym membership isn't an option… (P04).

This comment reveals the disjuncture between idealised care plans and socioeconomic or physical realities. Nurses recognised these constraints and viewed their role as negotiating these contradictions in ways that preserved both dignity and clinical benefit.

…you try to find the middle ground. You don't throw out the evidence, but you have to meet patients where they are. Otherwise, it's not care, it's a lecture (Nurse 09).

These accounts show that co‐production required navigating tensions between clinical ideals and the realities of patients' lives. Subthemes 5.3.1–5.3.3 reveal how successful co‐production rests on reconfiguring evidence as dialogic, situated and shaped through ongoing negotiation.

### 5.4 The Relational Foundations of Co‐Production

Beyond the integration of knowledge, participants consistently foregrounded the relational dimensions of co‐produced care. Co‐production was not experienced as a purely rational or procedural process. It was rooted in trust, empathy and emotional availability. Nurses and patients described the relationship as the medium through which shared decisions became possible, and the emotional and professional labour required to maintain such relationships as central to the experience.

#### 5.4.1 Trust as a Precondition for Engagement

Across both nurse and patient accounts, trust emerged as the foundation upon which co‐production was built. Without trust, attempts at shared decision‐making were described as shallow or performative. One nurse articulated this need for consistency and attentiveness:

…trust isn't given automatically. It's earned through every conversation, every moment you genuinely listen. Patients can tell when you're just going through the motions (Nurse 06).

This illustrates that trust is not a static condition but a process achieved over time. It is built through sustained emotional investment and attentiveness to the person behind the diagnosis. For patients, the experience of being listened to and remembered shaped their willingness to engage.

…at first, I didn't really speak up…I thought, ‘They're the expert […] but once I saw she [the nurse] actually listened and remembered the details of my life, I felt safe enough to be honest (P05).

These narratives highlight that co‐production is not simply about information exchange but about building relational safety. Trust enables honesty, and honesty allows for care plans that reflect real lives, not assumed norms.

#### 5.4.2 Relational Labour and Professional Vulnerability

For many nurses, practising relationally meant stepping out of the traditional expert role and allowing space for shared vulnerability. This shift was described not as weakness but as a deliberate professional stance.

…it's not easy to admit when you don't have all the answers. But I find that patients respond better when you show you're human too… (Nurse 02).

Here, professional vulnerability becomes a strength, creating space for co‐learning and mutual respect. Patients responded positively to this approach, interpreting it as a signal of respect and openness:

When a nurse says, ‘I'm not sure…let's figure it out together,’ it changes everything. You feel like it's a team, not a top‐down order (P09).

The emotional labour of maintaining this stance was acknowledged by nurses, who spoke of the effort required to balance empathy with professional obligations. This theme, taken together with 5.4.1, shows how co‐production rests not only on shared knowledge but on shared humanity. The capacity to trust, relate and be vulnerable formed the emotional scaffolding upon which meaningful care was built.

### 5.5 Organisational Pressures and Misalignments

While participants emphasised the centrality of relationships to effective co‐production, they also highlighted the significant constraints imposed by organisational structures. These pressures often created a mismatch between the values of co‐produced, person‐centred care and the realities of service delivery. Nurses and patients alike described how systemic conditions limited their capacity to engage meaningfully with one another.

#### 5.5.1 Time as the Missing Ingredient

Time emerged as one of the most frequently cited barriers to co‐production. Nurses spoke candidly about the limitations of tightly scheduled appointments and the pressure to deliver efficient care within strict timeframes. This sense of constraint disrupted their ability to build rapport, understand context and develop shared plans with patients.

…appointments are fifteen minutes. How do you build trust, have a real conversation, and make a shared decision in that time? It's impossible… (Nurse 08).

The emotional weight of this time pressure was not limited to professionals. Patients sensed when clinicians were rushing and described how it discouraged them from raising complex or sensitive issues.

You can feel it when they're rushing. It makes you think twice about bringing up anything complicated… (P01).

These accounts reveal how time scarcity not only hinders relational depth but also constrains patients' sense of agency, silencing the very voices that co‐production seeks to elevate. The relational labour described earlier in Theme 5.4 requires time and attentiveness, yet the organisational environment often offers little room for this kind of engagement.

#### 5.5.2 Metrics Over Meaning

A recurring tension was the prioritisation of measurable outcomes over relational and contextual quality. Several nurses expressed frustration with having to focus on targets rather than the person in front of them.

…you end up chasing numbers…blood pressure targets, cholesterol levels…but miss the person. The system doesn't measure trust or understanding… (Nurse 10).

This statement encapsulates a wider concern about the reduction of care to quantifiable indicators. While metrics can guide quality improvement, participants felt that excessive focus on them led to transactional interactions. Patients noticed this shift as well:

It's like being on a conveyor belt…quick check, tick the box, move on…there's no space for my story (P03).

Nurses and patients experienced emotional and ethical tension when system demands conflicted with the goals of relational care. This theme builds on earlier insights about relational foundations and illustrates how organisational cultures may inadvertently discourage the very practices they claim to support.

#### 5.5.3 Variability Across Settings

Experiences of co‐production were not uniform across health settings. Participants described how different organisational environments shaped what was possible in practice. One nurse compared her work in community and hospital settings.

…in the community clinic, we're given a bit more breathing room. In hospital, it's target‐driven…faster, faster, faster. You do what you can, but the structure makes it harder (Nurse 01).

Such contrasts suggest that co‐production is not simply a matter of personal will or clinical intent but is deeply shaped by institutional conditions. Patients noticed these differences too.

…some places you feel seen. Others, you're just another number in the system. It depends a lot on where you go and who you get (P06).

These observations reinforce the notion that co‐production is a situated practice. Organisational context can either facilitate or obstruct collaborative care, and variability between settings often determines whether co‐production is meaningful or superficial. This subtheme connects closely with earlier discussions of trust and vulnerability, illustrating how relational practices are always embedded in wider systemic dynamics.

### 5.6 Shifting Identities and Power Dynamics

Participants spoke about how the process of co‐production reshaped their understandings of power, expertise and responsibility. As patients became more active in their care and nurses adopted more facilitative roles, traditional professional boundaries began to shift. These evolving dynamics challenged conventional assumptions about who holds knowledge and authority in healthcare.

#### 5.6.1 Patients as Active Partners

Several patients reflected on a shift from passive recipient to active participant. They described a growing sense of involvement and ownership over care decisions, often contrasting current experiences with earlier, more hierarchical models.

…before, it felt like they just told you what to do […] now, they ask me what's realistic. It feels more like I have a say (P08).

Another elaborated on this transformation:

…I still respect their knowledge, but now I feel like my lived experience matters too. It's not just about following orders anymore… (P04).

These accounts reveal how co‐production can promote a sense of dignity and mutual respect. When patients feel that their expertise is valued, their willingness to engage increases. This theme connects to earlier discussions about evidence and lived experience, emphasising that shared decision‐making relies not just on technical knowledge but also on recognising patients' experiential authority.

#### 5.6.2 Nurses Redefining Expertise

From the perspective of nurses, co‐production involved a rethinking of professional identity. Many described moving from a stance of expert authority to one of facilitator and collaborator.

…I used to think my job was to have the answers. Now, I see it as helping the patient find what works for them. It's a different kind of expertise…one that's shared… (Nurse 07).

This shift was not always easy. Relinquishing control could feel uncomfortable, especially when organisational expectations still rewarded directive approaches. One nurse acknowledged this tension:

Sometimes it's hard to let go of being in control. But if we want genuine co‐production, we have to be willing to share the power (Nurse 05).

These narratives show that co‐production involves learning new skills and rethinking traditional professional roles. The emotional and ethical challenges described here resonate with those explored in Theme 5.7, where the complexity of navigating care together is brought into focus.

### 5.7 Emotional and Ethical Complexity in Co‐Producing Care

Participants drew attention to the emotional and ethical terrain of co‐producing care. Beyond technical decisions and shared plans, co‐production involved navigating feelings, values and tensions that emerged from the uncertainties and ambiguities of long‐term condition management.

#### 5.7.1 Navigating Emotional Landscapes

Both nurses and patients acknowledged that chronic illness is not only a physical condition but also an emotional experience. Addressing these emotional dimensions was seen as central to meaningful care.

…you're not just dealing with blood sugars or blood pressure…you're dealing with fear, frustration, hope. Ignoring that would be bad care (Nurse 04).

Patients expressed appreciation when their emotional world was acknowledged alongside their physical symptoms:

…it's not just about what's happening physically. The emotional part…the fear, the fatigue…it matters too. When they [nurses] get that, it changes everything (P02).

These reflections demonstrate that emotional labour is not optional but intrinsic to co‐production. Ignoring emotional needs risks undermining trust and diminishing the quality of relational care. This theme builds on earlier subthemes around relational labour and underscores the need for systems that support emotional attentiveness.

#### 5.7.2 Ethical Tensions in Shared Decision‐Making

Participants also described ethical tensions that arose when patients' choices conflicted with clinical recommendations. Nurses spoke about the difficulty of respecting autonomy while feeling accountable for health outcomes.

…there are times when I know the evidence says one thing, but the patient's choice goes another way. It's hard, but respecting that choice is part of the partnership… (Nurse 11).

Patients acknowledged the weight of these decisions but valued having the freedom to choose.

…it's not that I ignore the advice…I listen…but in the end, it's my life. I'm the one living it day to day (P05).

This subtheme highlights the ethical complexity of co‐produced care. Mutual respect does not eliminate disagreement, but it does provide a foundation for navigating difference. These insights bring together multiple strands from earlier themes: The balancing of different knowledges, the trust required for vulnerability and the organisational contexts that constrain or enable these conversations.

## 6 Discussion

This study explored how nurses and patients experience and make sense of co‐producing care in the context of managing LTCs, and how relational, emotional and organisational factors influence the use of EBP in these encounters. The findings offer a rich, idiographic account of how co‐production is experienced at the intersection of formal evidence, lived experience and organisational realities. This study adds to existing literature that sees co‐production as more than a technical process (Boulton et al. 2017; Greenhalgh et al. 2016). Participants described it as relational and moral, shaped by trust, emotion and structural pressures.

Participants spoke of EBP not as a static or universal process but as a situated negotiation. Nurses used guidelines as starting points rather than prescriptive rules, recognising that patients' lives often exceeded the scope of formal recommendations. This finding echoes earlier research highlighting the need for clinical guidelines to be adapted to the individual and social realities of patients' lives (Barry and Edgman‐Levitan 2012; Brown et al. 2018; Locock et al. 2014; May et al. 2014). Similarly, patients valued when their embodied knowledge was acknowledged as a valid form of evidence. Prior work in chronic illness care has emphasised the importance of experiential knowledge, particularly in conditions requiring daily self‐management (Pols 2013; Upshur and Tracy 2013).

The first research question asked how nurses and patients experience and make sense of co‐producing care in the management of LTCs. Participants described co‐production as grounded in relationships, emotional resonance and mutual respect, rather than as a purely instrumental process. Trust was central. When patients felt listened to and known as individuals, they were more willing to participate in decision‐making. This aligns with previous findings that trust and continuity are key facilitators of shared decision‐making, particularly for marginalised populations (Madden and Speed 2017; Rycroft‐Malone et al. 2008). Relational labour was also evident in the accounts of nurses, who spoke of the vulnerability involved in acknowledging uncertainty and sharing control. These findings reinforce the argument that co‐production requires emotional skill and moral courage (Brown et al. 2018; Kitson et al. 2013; Horne et al. 2020).

The second research question explored how evidence is negotiated in co‐produced encounters. Participants described the tensions that emerged when clinical recommendations conflicted with patients' lived realities. Nurses engaged in a process of translation and compromise, aiming to integrate guideline‐based evidence with individual needs and preferences. This resonates with research on contextualised EBP, which highlights the need for practitioners to navigate conflicting demands and forms of knowledge (Kitson et al. 2013; Rycroft‐Malone et al. 2008). Patients expressed a desire for their experiential knowledge to be respected, especially in managing the practical and emotional demands of daily life with chronic illness. The inclusion of patients' narrative and embodied knowledge has been recognised as critical to person‐centred care, yet remains inconsistently embedded in clinical practice (Entwistle and Watt 2013; Parke et al. 2020).

Frameworks were not used to structure the analysis but were reflexively engaged in the interpretive phase. McCormack and McCance's Person‐Centred Nursing Framework helped illuminate participants' emphasis on dignity, empathy and relationship‐building as central to co‐production (McCormack and McCance 2006). Similarly, Rycroft‐Malone's Contextual Model of EBP was useful for understanding how structural constraints, such as time pressure and performance metrics, shaped the possibilities for co‐produced care (O'Brien et al. 2014). These frameworks provided a language for articulating the moral and organisational tensions revealed in the data but did not determine the analytic codes or themes (Moulton et al. 2016; Svenaeus 2001; Toombs 1993). This analytic stance preserved fidelity to the phenomenological orientation of the study.

A phenomenological lens also allowed attention to the subtle textures of participants' experiences, the pauses, tensions and affective tones that shaped how co‐production was lived. For example, several patients described the emotional labour of negotiating clinical authority, while nurses spoke about the discomfort of relinquishing control. These nuanced accounts point to the emotional demands of shared work and the fragility of trust within structurally constrained systems (Ominyi, Eze, et al. 2025; Ominyi, Ukpai, et al. 2025). Prior phenomenological studies of chronic illness and care relationships have similarly emphasised the emotional complexity of partnership (Toombs 1993).

## 7 Strengths and Limitations

This study provides a detailed, idiographic account of how co‐production and EBP are experienced in long‐term condition management, capturing both nurse and patient perspectives. One of the study's strengths lies in the use of IPA, which facilitated a deep engagement with participants' meaning‐making processes. The analysis was conducted with close attention to linguistic nuance and emotional tone, allowing for a layered interpretation of how relational, contextual and evidentiary elements interplayed in care encounters. The co‐design of the interview guides with PPI contributors added credibility and ethical robustness to the research process, enhancing the relevance and sensitivity of the questions. Triangulation between patient and nurse narratives strengthened interpretive depth and offered insights into the relational dynamics of co‐produced care.

However, the study also has limitations. The sample comprised 20 participants drawn from a single geographical area, which may influence the transferability of findings. While IPA does not seek statistical generalisability, it prioritises depth, contextual richness and idiographic insight (Eatough and Smith 2017). The interpretations presented here are, therefore, grounded in the particularities of participants' lived experiences within specific service configurations, cultural norms and clinical practice contexts (Greenhalgh et al. 2016). These situated accounts offer valuable insights but should be understood as contextually bound rather than universally representative. A more diverse sample may have yielded different emphases, particularly in relation to ethnicity, socioeconomic status or other intersecting identities. Phenomenological inquiry, while powerful in revealing depth and complexity, also has limitations in capturing systemic structures and macro‐level influences (Smith and Nizza 2022). The focus on individual narratives means that broader social determinants of health may not be as fully developed. However, this limitation is also a strength, as it centres the lived realities that are often flattened or marginalised in large‐scale studies (Gharaveis et al. 2021).

## 8 Implications for Practice, Policy and Research

Findings from this study suggest several implications for nursing practice. First, co‐production should be understood not merely as a technique or intervention, but as a relational and ethical practice that requires time, trust and emotional engagement. Clinical training programmes could better prepare nurses for this work by including communication skills, reflective practice and collaborative decision‐making as core competencies. Second, organisational structures need to support, rather than constrain, relational practice. Time pressures, performance metrics and rigid guidelines often inhibit the flexibility required for co‐produced care. Policy frameworks should recognise the value of narrative, emotion and context in shaping clinical outcomes. There is scope for service redesign to create conditions in which trust and continuity can flourish, especially in primary care and community‐based settings.

For research, the study highlights the need for more relational approaches to understanding EBP. Quantitative metrics alone are insufficient for capturing the lived realities of co‐production. Future studies could explore the use of participatory or longitudinal qualitative methods to further examine how co‐production unfolds over time, particularly in under‐researched populations or contexts. Methodologically, the study illustrates the value of IPA for surfacing the affective, moral and relational dimensions of practice that are often missed in standard evaluations. At the same time, it calls for creative integration with conceptual frameworks that can illuminate organisational and structural forces without diluting the idiographic focus.

## 9 Conclusion

This study provides new insights into how nurses and patients experience the co‐production of evidence‐based care in managing long‐term conditions. Rather than viewing evidence as fixed or universal, participants described care as a negotiated process shaped by relational, emotional and organisational dynamics. Trust, mutual respect and contextual sensitivity were foundational to meaningful co‐production. The findings affirm the importance of attending to the human dimensions of healthcare, particularly in contexts where standardised practices risk displacing lived experience.

Conceptual frameworks such as McCormack and McCance's Person‐Centred Nursing Framework and Rycroft‐Malone's Model of EBP proved useful in interpreting the interplay between care relationships, evidence and structure. However, their application was most meaningful when used to reflect on and deepen emerging themes rather than dictate analysis. Phenomenological inquiry remains a powerful tool for capturing these nuances, though its limits in addressing wider structural determinants must be acknowledged. The findings point to the need for practice environments that value relational care and recognise the multiplicity of knowledge forms. Supporting nurses and patients in this work requires organisational commitment, conceptual flexibility and methodological openness to the complexity of real‐world care.

## Author Contributions

**J.O**., **A.C**. and **U.C**. contributed to the conceptualisation of the study, including the formulation of research aims and overall design; all three authors were involved in the development of the methodological approach and the integration of theoretical frameworks. **J.O**. and **A.C**. conducted the investigation, including participant recruitment and data collection; **J.O**. led the data curation process, managing transcription, anonymisation and organisation of interview data. **J.O**. also led the formal analysis; **A.C**. and **U.C**. contributed to validation through independent coding of selected transcripts and collaborative theme refinement. Project administration was coordinated by **J.O**., who managed timelines, ethics approvals and site engagement. **A.C**. and **U.C**. provided ongoing supervision and guidance throughout the research process. **J.O**. prepared the original draft of the manuscript; **A.C**. and **U.C**. contributed to writing – review and editing by offering critical intellectual input and editorial revisions. **J.O**. developed visual elements such as conceptual figures and tables. All authors contributed resources to support the study, including site access and participant engagement strategies. All authors read and approved the final manuscript and agreed to be accountable for all aspects of the work, ensuring that any issues related to the integrity or accuracy of the research are appropriately addressed.

## Ethics Statement

The study was conducted in accordance with the guidelines of the Declaration of Helsinki and was approved by the University of Beds Research Ethical Approval (Reference ID: UoB/00184; Date of approval: 07.09.22), ensuring compliance with national regulations. Any data utilised in the submitted manuscript have been lawfully acquired in accordance with The Nagoya Protocol on Access to Genetic Resources and the Fair and Equitable Sharing of Benefits Arising from Their Utilisation to the Convention on Biological Diversity. The relevant fieldwork permission was obtained and listed the permit numbers.

## Consent

Informed consent to participate was obtained from all participants prior to their enrolment in this study. Participant information sheets and consent forms were provided in advance via email. These documents were then reviewed and verbally confirmed at the start of each observation episode and interview recording.

## Conflicts of Interest

The authors declare no conflicts of interest.

## Data Availability

The data that support the findings of this study are available from the corresponding author upon reasonable request.
